# Association between sleep and physical activity data, and depressive symptoms in Thai elderly

**DOI:** 10.1371/journal.pone.0329978

**Published:** 2025-08-14

**Authors:** Hathairat Kosiyaporn, Jiranun Thiphong, Orratai Waleewong, Rujira Adhibai, Supika Cheujew, Nisachol Cetthakrikul, Sopit Nasueb, Kamolphat Makchang, Yanisa Pumsutas, Patjirapohn Suosot

**Affiliations:** International Health Policy Program, Ministry of Public Health, Nonthaburi, Thailand; University of Sao Paulo, BRAZIL

## Abstract

**Background:**

Geriatric depression often goes unnoticed due to recall bias and overlapping symptoms with normal aging by using questionnaire screening tool. Consequently, this study aims to explore the associations between passive sensing parameters, such as physical activity and sleep characteristics collected from smart devices, and depression screening scores, aiming to validate its efficacy as a detection tool for the Thai elderly.

**Methods:**

The prospective cohort study was conducted from July to September 2023. One hundred and seventy-seven elderly individuals were purposefully selected from the main districts of each province across five regions in Thailand. Inclusion criteria required participants to be aged 60 years or older, socially active, and free from diagnoses of cognitive impairment and mental health disorders. Participants were required to wear an Actigraph wGT3X-BT collecting data on physical activity and sleep characteristics. The Patient Health Questionnaire (PHQ-9) was used to screen for depression every two weeks. Univariate and multivariate regression analyses were performed to identify association between passive sensing parameters and PHQ-9.

**Results:**

There is an association between physical activity parameters and depression score. A One- unit increase in Vector Magnitude in Counts per Minute (VM CPM) and step count, PHQ-9 score would be statistically significant reduced by 0.001 and 0.00007 to 0.00008 score over both two-week periods (p-value <0.1). Only Wakefulness After Sleep Onset (WASO) of all sleep variables showed an association with PHQ-9 in univariate analysis but it did not show further relationship after adjusting with baseline PHQ-9 score and other confounders. In addition, participants who lived outside Bangkok and being younger were more likely to have lower PHQ-9 score.

**Conclusion:**

There is a potential to apply passive sensing data in mental health issues in Thailand. However, more evidence is needed to ensure the accuracy of sensor detection and appropriate algorithm to predict depression. Moreover, the implication of passive sensing data from smart devices supporting public health policy should be explored.

## Introduction

Thailand has transitioned into a ‘complete aging society.’ By 2020, the number of Thai elderly individuals (aged 60 years and older) had reached 18% of the Thai population, which totals 66.5 million [1]. With advancing age, physical deterioration can lead to multiple diseases and health problems; however, mental health is often neglected compared to physical illnesses [2]. In Thailand, one-third of the elderly population is mentally vulnerable, and depression is among the five most common mental illnesses in this age group [3]. In addition, geriatric depression is often overlooked, under-recognized, and undetected as a result of complexities and limited access to questionnaire-based screening. Depressive symptoms in older adults can also be attributed to normal aging and/or physical illnesses, which both patients and clinicians tend to misinterpret [4]. For instance, the overlapping depressive symptoms and normal aging includes loss of appetite, insomnia, psychomotor retardation, and forgetfulness [5]. History taking and self-assessment responses may be biased by memory issues, a common occurrence in the elderly, especially those with cognitive impairments [6,7]. Accordingly, screening questionnaire for depression may not fully coverage in all elderly. A data from Department of Mental Health (Ministry of Public Health; MOPH) found that only 68% of the elderly were screened using the 2Q questionnaire in 2022 (Thai version of the Patient Health Questionnaire-2; PHQ-2) [8]. Thus, the unmet needs existed there is a challenge in screening depression in elderly by using questionnaires.

Due to subjective, time-consuming, and challenging nature of traditional screening tools leading to poor access to these activities, passive sensing with smartphones and wearables (devices that track daily activities) are part of a rapidly growing area in pervasive health care [9]. Passive sensing quantifies moment-to-moment behaviors by collecting a broad spectrum of physiological and behavioral data from sensors on connected devices in real-life settings. This method ensures objectivity, accuracy, and ecological validity in the data collection with minimal user interactions [9,10]. Passive sensing data collection also has the potential to be a more accurate and less burdensome approach to detection when compared with traditional paper-based screening tools [11]. In Thailand, individuals aged 60–70 exhibit moderate digital literacy, while the near-elderly group (50–59 years old) demonstrates a high level of proficiency [12,13]. A survey by the NSO revealed an increasing trend in mobile phone usage among Thai individuals aged >60, rising from 12% in 2016 to 20% in 2022, with approximately 11% using smartphones. Additionally, Thailand’s wearables market experienced a 12.6% growth from 2019 to 2020 [14,15]. It is speculated that the number of elderly individuals with digital access and literacy will continue to rise in the future. Hence, it is possible that passive sensing data from smartphones and smartwatches can potentially fill the above-mentioned gap in the elderly population.

In a systematic review of the literature on using passive sensing data in smartphones or smartwatches for screening depression in the elderly from 2012 to 2022, we found that 16 out of 21 studies were conducted in Western countries and the most popular passive sensing data were related to sleep and physical activity measured [16]. Regarding sleep characteristics, Wakefulness after sleep onset (WASO) was found to be related to depressive symptoms with a potential exacerbating effect over time when the duration of WASO exceeded one hour [16–18]. On the other hand, other sleep characteristics such as sleep efficiency, sleep latency, and long wake episodes yielded mixed results in terms of statistical significance. The physical activity has a negative relationship with depressive symptoms; for example, a study showed that moderate-to-vigorous physical activity (MVPA) could reduce depression symptoms [19]. Moreover, it is necessary to more explore various parameters in the measurement of physical activity such as step count to ensure a comprehensive data collection. Thus, passive sensing data for both sleep, and physical activity parameters can be a potential tool for depression detection.

Nevertheless, there is still a lack of literature on the use of passive sensing data for screening depressive symptoms in the elderly, especially in a low- and middle-income context like Thailand [20]. Based on existing evidence of relationship between passive sensing data (such as physical activity and sleep characteristics) and depressive symptoms, this study aims to identify association between passive sensing parameters and depression screening scores in Thai elderly individuals. The findings will be used to develop a valid detection tool for depression in the elderly. Moreover, the technology acceptance will be explored to ensure acceptability and feasibility of device usage in the future.

## Materials and methods

This prospective cohort study was conducted from July to September 2023. The study received ethical approval from the Ethics Committee of the Institute for the Development of Human Research Protections (HSRI 809/2565). All methods were carried out in accordance with relevant guidelines and regulations. All participants were informed about the study and provided written consent before the research team commenced the project.

### Data collection

A sample size of 183 participants was calculated using a correlation formula for an infinite population [21]. Participants were purposively selected from provinces with limited access to elderly depression treatment in each region of Thailand [22]: Bangkok (Capital), Saraburi (Central), Loei (North-Eastern), Phuket (Southern), and Lampang (Northern). Elderly clubs in the city districts of each province were purposively selected, and 40 elderly individuals from each club were recruited through quota sampling. Inclusion criteria required participants to be aged 60 years or older, socially active, and free from cognitive impairment and mental health disorders, except for depression. Participants who did not complete the follow-up were excluded from the study. The Final analysis included177 participants.

### Data measurement

Participants were required to wear an Actigraph wGT3X-BT, a wrist-worn device designed to collect movement data in three axes, enabling the identification of physical activity and sleep characteristics [23]. This device had to be worn on the non-dominant wrist for a duration of one month, with the recommendation to wear it at all times, except during water-related activities such as bathing or washing. The physical activity variables was activity count calculated to variables such as Vector Magnitude (VM: summary of three axis activity count in square root), step counts, and total time in each physical activity level (sedentary, light, moderate, vigorous), while sleep characteristics were sleep and wakefulness duration calculated to parameters such as Total Sleep Time (TST), Wakefulness After Sleep Onset (WASO: the total minutes of wakefulness after sleep onset), Sleep Efficiency (SE: the percentage of sleep minutes relative to the total number of minutes the subject was in bed), and Sleep Fragmentation Index (SFI: the percentage and reflecting restlessness during the sleep period) [24–26]. At the beginning of the study, demographic information was collected, including age, gender (male/female), education (primary education/secondary education/diploma/university and higher), occupation (unemployed or retired/self-employed/freelance/agriculture/public employee), marital status (single/married/ divorce or widow) and income. Additionally, other factors associated with depression were recorded, such as family members (alone/with parents or spouse or grandchildren only/with mixed generations), alcohol and tobacco use, drug use (yes/no), comorbidities (none/cardiovascular-related diseases/orthopedic and hormonal related diseases/mental illness), and medical history (none/having drugs affecting sleep and movement), were recorded. The depression screening process was conducted every two weeks (week 2 and 4), using the PHQ-9 questionnaire, which consists of nine questions related to depressive symptoms ranging from 0 to 27 points [27]. Participants with mild to severe depression scores and those expressing suicidal thoughts were referred for further treatment. At the end of data collection, a technology acceptance survey adapted from Puri et al. (2017) were administered to gauge participants’ perceptions of this device. This assessment covered the aspects: ease of use, equipment characteristics, privacy concerns, facilitating and barrier conditions, subjective norms, and intentions to use, using a Likert scale ranging from 1 (strongly disagree) to 5 (strongly agree) [28].

### Data analysis

The valid wearing time was considered if it was worn for more than four days within a week and for more than 10 hours per day [29]. Non-wear time was identified and subsequently excluded from the analysis. The study period was classified into a first half and second half of two-week period.

Physical activity and sleep parameters were categorized as independently continuous variables. Physical activity was assessed by employing the method described by Freedson et al. (2011) to determine the summary of average Vector Magnitude in Counts per Minute (VM CPM), and Daily Step Counts [26]. Sleep characteristics were evaluated selecting specific metrics: WASO, SE, SFI [24]. All independent variables were calculated in mean of each two-week period. The PHQ-9 score was continuous variable defined as a dependent variable. Changes in this score were analyzed every two weeks (weeks 2 and 4) compared to the baseline PHQ score at the beginning. Confounding variables included age, gender, education, occupation, marital status, income, province, household members, alcohol use, tobacco use, drug use, comorbidities, and medical history, which were analyzed as categorical variables, as detailed in Table 1.

**Table 1:** Demographic characteristics of participants.

Demographic characteristics	Number	Percentage
**Province**		
Lampang (Northern)	41	23.1
Phuket (Southern)	35	19.8
Bangkok	38	21.5
Saraburi (Central)	31	17.5
Loei (North-Eastern)	32	18.1
**Gender**		
Female	150	84.7
Male	27	15.3
**Education**		
Primary education	73	41.3
Secondary education	19	10.7
Diploma	45	25.4
University and higher	40	22.6
**Occupation**		
Unemployed/Retired	116	65.6
Self-employed	39	22.0
Freelance	19	10.7
Agriculture	2	1.1
Public employee	1	0.6
**Marital status**		
Single	13	7.3
Married	97	54.8
Divorce/widow	67	37.9
**Household members**		
Alone	20	11.3
With parents/spouse/grandchildren only	40	22.6
With mixed generations	117	66.1
**Current smoking**		
No	174	98.3
Yes	3	1.7
**Current alcohol used**		
No	157	88.7
Yes	20	11.3
**Current drug used**		
No	168	94.9
Yes (e.g., Cannabis)	9	5.1
**Comorbidity (physical illness)**		
No	27	15.3
Cardiovascular-related diseases	107	60.4
Orthopedic and Hormonal related diseases	43	24.3
**Comorbidity (mental illnesses)**		
No	167	94.3
Yes (Other mental illness)	7	4.0
Yes (Sleep and eating disorder)	2	1.1
Yes (Others)	1	0.6
**Medication**		
No	162	91.5
Having drugs affecting sleep and movement	15	8.5
**Total**	**177**	**100.0**

The demography of participant was descriptively analyzed in minimum, median, interquartile range (IQR), and maximum values among continuous variables and was classified as category (see Table 1). Inferential analysis involved the application of linear regression to identify associations between each physical activity and sleep characteristics parameter (dependent variables) and the change of PHQ-9 scores (independent variables) during the first (week 0–2: compared round 2 with round 1) and second half (week 2–4: compared round 3 with round 1) of study. It was adjusted for confounding variables such as age, gender, education, occupation, marital status, income, family members, alcohol and tobacco use, drug use, comorbidities, and medical history (confounding variables). Only variables that showed statistical significance in the univariate analysis and identify association in previous literature were included in the multivariate analysis at a 95% level of statistical significance.

## Results

### Demographic characteristics of participants

**Fig 1:**
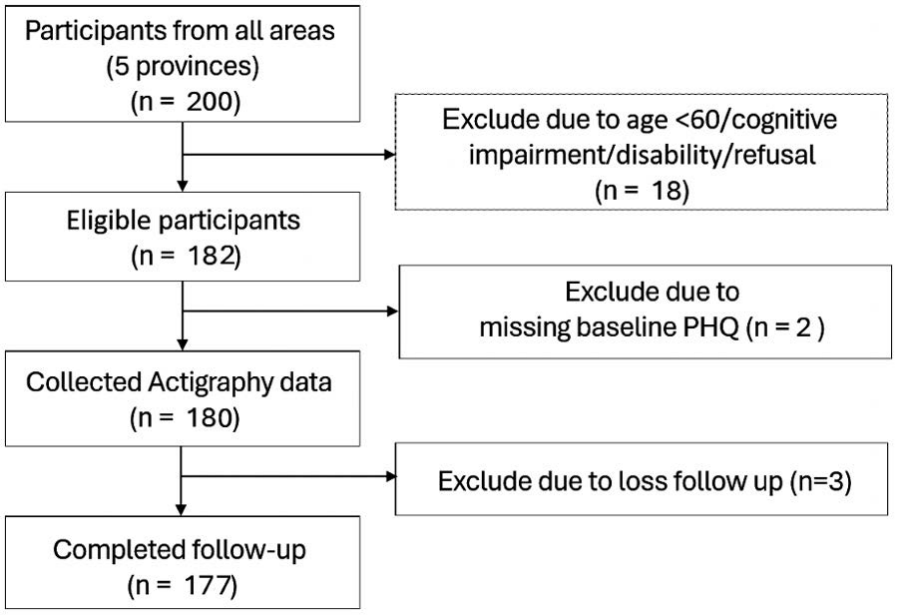
Flow of recruited participants.

A total of 177 Thai elderly participants were included in the study, with nearly equal distribution across all regions (see Fig 1). The highest proportion of participants was in Northern region and the lowest proportion were in North-Eastern and Central regions, accounting for 23.1% and 18.1%, respectively (see Table 1). The majority of participants were women (84.7%), with a median age of 68 years (min = 60; IQR = 8.8; max = 85). Most participants had a primary education level (41%) and were either unemployed or retired (65.6%). The median monthly income was 9,500 Baht (approximately 270 USD) (min = 0; IQR = 21,500; max = 300,000). More than half of the participants were married (54.5%), followed by those who were divorced or widowed (37.9%). Regarding household composition, participants typically lived with mixed generations, such as children and grandchildren (66.1%).

Regarding health risk behaviors, a large proportion of participants reported not smoking (98.3%), abstaining from alcohol consumption (88.7%), and refraining from drug use (94.9%) in the past 12 months (see Table 1). Notably, a significant number of participants had cardiovascular diseases (CVD), such as hypertension, hyperlipidemia, diabetes, and heart disease (60.4%) but only 8.5% took regular medications that affected sleep and movement. Moreover, most participants (94.3%) reported no history of mental illness.

### PHQ-9 score of participants during the study period

**Fig 2:**
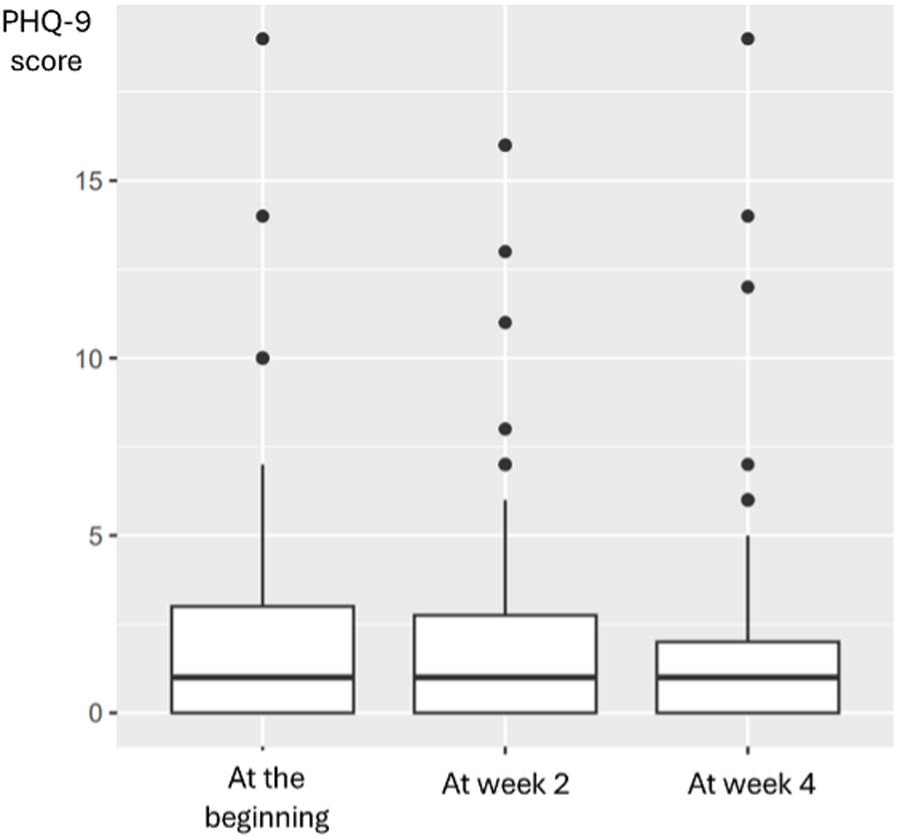
Summary of PHQ-9 score at week 0, 2 and 4.

The median PHQ-9 score at the beginning was one (min = 0; IQR = 3; max = 19), which remained the same at week 2 (min = 0; IQR = 2.75; max = 16) and week 4 (min = 0; IQR = 2; max = 19), as shown in Fig 2.

### Physical activity and sleep parameters of participants during the study period

**Fig 3:**
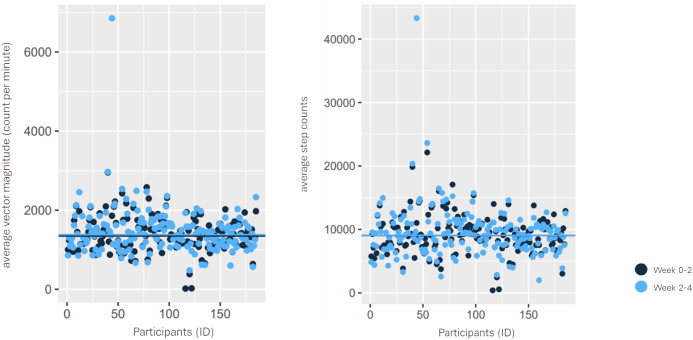
Summary of average vector magnitude (count per minute) and step counts during weeks 0-2 and week 2-4.

The median average vector magnitude (counts per minute) during weeks 0–2 was 1,337 (min = 17; IQR = 632; max = 2,953). During weeks 2–4, the median of an average vector magnitude (count per minute) marginally increased accounting for 1,372 (min = 479; IQR = 638; max = 6,853). The median of average daily step count during weeks 0–2 was 8,995 (min = 407; IQR = 4,576; max = 22,134). During weeks 2–4, the median of average step counts slightly increased accounting for 9,032 (min = 2,008; IQR = 4,621; max = 43,292), see Fig 3.

**Fig 4:**
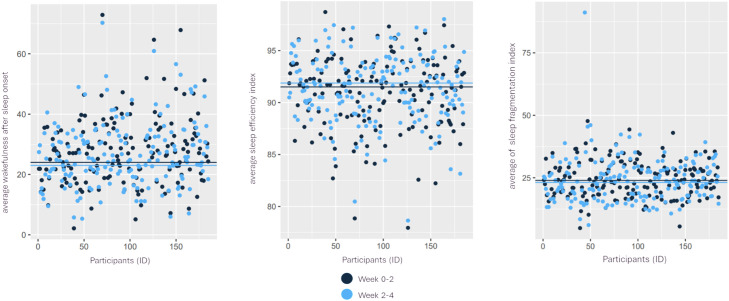
Summary of average wakefulness after sleep onset (WASO), sleep efficiency and sleep fragmentation index during weeks 0–2 and weeks 2–4.

The median of average minutes during wakefulness after sleep onset (WASO) during weeks 0 to 2 was 24 minutes (min = 2; IQR = 25; max = 73). During weeks 2 to 4, the median of average minutes during wakefulness after sleep onset reduced to 23 minutes (min = 5; IQR = 25; max = 70). In addition, the median of the average sleep fragmentation index from weeks 0 to 2 was 23.7 (min = 5; IQR = 21.8; max = 48) which decreased to 23.0 (min = 6; IQR = 22.3; max = 91) in the latter period. The median of average sleep efficiency during weeks 0 to 2 was 91.6% (min = 78; IQR = 7.2; max = 99) and it slightly increased to 92.0% (min = 79; IQR = 7.1; max = 98) in weeks 2 to 4, (see Fig 4).

### Association between physical activity/sleep parameters and PHQ-9 score

After removing missing data, there were 166 participants in the first round and 176 participants in the second round. The variables significantly associated with PHQ-9 score in univariate analysis from both rounds were included in the multivariate analysis, see S1 Table.

In multivariate analysis, for each one-unit increase in VM CPM and step count, the PHQ-9 score would be statistically significant reduced by 0.001 score and 0.00007 to 0.00008 score in both round period (p-value <0.1) (see Tables 2 and 3). This indicates that higher levels of physical activity were associated with lower depression scores. In addition, participants who lived outside Bangkok and those who were younger were more likely to have lower PHQ-9 score but only in the first round of study (see Tables 2–4). However, WASO showed no association with PHQ-9 score (see Table 4).

**Table 2:** Multivariate analysis of between VM CPM and PHQ-9 score (Round 2 and 3) adjusted with confounders.

Independent/confounding variables	PHQ-9 score (round 2 compared with round 1)			PHQ-9 score (round 3 compared with round 1)		
	Correlation coefficient	95% confident interval	P-value	Correlation coefficient	95% confident interval	P-value
VM CPM	−0.001	0.000	0.062*	−0.001	0.000	0.063*
Province (vs BKK vs non BKK)	−0.903	0.364	0.014**	−0.279	0.368	0.449
Age	0.040	0.022	0.071*	0.007	0.022	0.754
Education (primary & secondary vs diploma & university)	−0.080	0.285	0.780	−0.074	0.282	0.793
Marital status (single/divorce/widow vs married)	−0.436	0.267	0.104	−0.227	0.267	0.396
Current smoking (no vs yes)	1.479	1.013	0.146	−0.646	1.054	0.541
Mental illnesses (no vs yes)	0.976	0.601	0.106	0.352	0.562	0.532
IADL (dependent vs independent)	−0.029	0.481	0.952	−0.642	0.487	0.189

Note: *confidence interval at 90%; **confidence interval at 95%.

**Table 3:** Multivariate analysis of between daily step count and PHQ-9 score (Round 2 and 3) adjusted with confounders.

Independent/confounding variables	PHQ-9 score (Round 2) (round 2 compared with round 1)			PHQ-9 score (Round 3) (round 3 compared with round 1)		
	Correlation coefficient	95% confident interval	P-value	Correlation coefficient	95% confident interval	P-value
Daily step count	−0.00008	0.00005	0.070*	−0.00007	0.00004	0.098*
Province (vs BKK vs non BKK)	−0.931	0.366	0.012**	−0.286	0.370	0.441
Age	0.041	0.022	0.064*	0.007	0.022	0.750
Education (primary & secondary vs diploma & university)	−0.044	0.282	0.876	−0.039	0.281	0.890
Marital status (single/divorce/widow vs married)	−0.437	0.267	0.104	−0.235	0.267	0.380
Current smoking (no vs yes)	1.466	1.015	0.151	−0.655	1.059	0.537
Mental illnesses (no vs yes)	0.958	0.600	0.113	0.326	0.563	0.564
IADL (dependent vs independent)	−0.101	0.478	0.833	−0.686	0.486	0.161

Note: *confidence interval at 90%; **confidence interval at 95%.

**Table 4:** Multivariate analysis of between WASO and PHQ-9 score (Round 2 and 3) adjusted with confounders.

Independent/confounding variables	PHQ-9 score (Round 2) (round 2 compared with round 1)			PHQ-9 score (Round 3) (round 3 compared with round 1)		
	Correlation coefficient	95% confident interval	P-value	Correlation coefficient	95% confident interval	P-value
WASO	−0.020	0.012	0.105	−0.004	0.013	0.766
Province (vs BKK vs non BKK)	−0.863	0.364	0.019**	−0.177	0.367	0.630
Age	0.038	0.023	0.098*	0.012	0.023	0.602
Education (primary & secondary vs diploma & university)	0.011	0.280	0.969	0.007	0.282	0.979
Marital status (single/divorce/widow vs married)	−0.433	0.267	0.108	−0.240	0.269	0.373
Current smoking (no vs yes)	1.666	1.009	0.101	−0.422	1.058	0.690
Mental illnesses (no vs yes)	0.833	0.600	0.167	0.315	0.569	0.581
IADL (dependent vs independent)	−0.109	0.479	0.820	−0.732	0.492	0.139

Note: *confidence interval at 90%; **confidence interval at 95%.

## Discussion

In this study, it demonstrated an inverse relationship between physical activity and depression severity as shown in a result from multivariate analysis that one-unit increase in VM CPM reduced depression scores by 0.001, and one-unit increase in step count decreased depression scores by 0.00007–0.00008. On the other hand, there was no association between sleep characteristics and depression as indicated by WASO showing no correlation with PHQ-9 scores. Additionally, during the first round only, younger participants and those residing outside Bangkok tended to have lower depression scores.

There is evidence of association between physical activity parameters and depression. The results of this study is similar to a study in elderly by O’Brien et al. (2017) and Gruenenfelder-Steiger et al. (2017) showing that the physical activity in a form of movement data like acceleration magnitude was considerably lower (t = 3.63; p = 0.001) with depression when compared to the control group [30] and an increase of one unit in physical activity unit of standardized daily step count) was associated with a 14 percent decrease of daily depressive mood within-person [31]. Physical activity is proved as protective measure of depression from a meta-analysis of cohort studies up to 2017. It found that individuals with high levels had lower odds of developing depression compared with people with low levels of physical activity (adjusted odds ratio = 0.83; 95% CI = 0.79–0.88; I2 = 0.00) [33]. In addition, late-life depression (depressive disorder at older age) represents somatic symptoms predominantly compared to mood symptoms [34]. Therefore, physical activity reduction has a potential to be a trigger of depressive symptoms in elderly.

Sleep characteristics are not associated with depressive symptoms in this study, while it has been proved a relationship between these variables in previous study. A systematic review of the association between passive sensing data and depression in the elderly by Adhibai et al. (2023) found that sleep parameters appear to be promising proxies for depressive symptoms, especially sleep characteristic variables, such as WASO [16] as same as a study by Franzen and Buysse (2008) found the bidirectional associations between sleep disturbance (especially insomnia) and depression [32]. Although this study reveals that WASO is related to PHQ-9 scores in univariate analysis, there is an inconsistent after adjusting for other confounders. It is important to note that not all sleep parameters are found to be related to depressive symptoms in the elderly, such as total sleep time, sleep latency, and sleep efficiency, which may need further study to explore these relationships.

Other confounding variables that seem to associate with depression is area of living. Those who live in Bangkok are more likely to have higher depressive score compared to people live in rural area. A meta-analysis study by Xu et. al (2023) showed that urban residence was significantly associated with a higher prevalence of depression in developed countries (OR = 1.30; 95% CI = 1.17–1.46; *z* = 4.75; *p* < 0.001) [33]. Moreover, age appears to have a potential impact on depressive symptoms. A systematic review and meta-analysis about the prevalence and determinants of depression among old age by Zenebe et al. (2021) revealed that socioeconomic factors such as age older than 75 years associated with depression in elderly populations [34]. However, the results from this study are not consistent throughout the study period, as the association is observed only in one round of the study. Therefore, further studies in specific contexts need to be conducted in the future.

There is ongoing effort to promote smart devices as detection tool for depressive disorder such as smartphone or wearable devices. Wearable devices have been one of the technologies used for detecting and predicting depression as these can collect and analyze biomarkers or biosignals such as heart rates, physical activities, sleep patterns and quality, blood oxygen, and respiratory rate [35] which is more popular especially in elderly [36]. It has been promoted as detection tool for depression, but it should be used in conjunction with other data sources like neuroimaging data and methods for diagnosing and predicting depression such as self-report questionnaires or interviews, to provide a more comprehensive understanding of a patient’s condition [35]. According to previous studies about feasibility of wearable devices, the participants reported high satisfaction, stating the devices were easy to use, helpful for setting goals, motivational, and useful for self-monitoring [36,37]. However, the evidence is needed to ensure accuracy of sensors and algorithms, and data integration to be appropriate screening and monitoring tools.

Although this study has strengths as the first study of passive sensing data and depression in Thailand, it also has several limitations. Firstly, the sample selection may be biased due to quota sampling across regions. It is a non-probability sample where participants in each region did not have equal probability of being selected, which might affect the representativeness of the sample. Secondly, participants had to remove the Actigraph during water-related activities, which might have impacted the validity of the wearing time. However, we asked participants to wear this device as soon as possible after they finished water-related activities, and all participants had sufficient wearing time for evaluation (more than 10 hours per day and 4 days per week). Lastly, the study period was short, so it could not capture significant changes in physical activity and sleep parameters, including PHQ-9 scores. Further studies should be conducted over a longer period, include participants with a broader range of PHQ-9 scores, such as patients, and apply devices that participants use in their daily lives such as smartwatches.

In Thailand, there is a potential to apply passive sensing data in mental health issues but there are technical and operational challenges in population scale. Sensing technology in mental health has been developed in various ways but only certain sensors are selected like DMIND application, developed by the Faculty of Medicine and the Faculty of Engineer, Chulalongkorn University, which is an application used for depression screening through facial and voice detection [38]. Other sensor variables used for depression detection such as sleep, physical activity, mobility, and socialization (e.g., call, text) [6], are less focused in Thailand. Smart devices, especially smartwatches, are capable of collecting these types of data; however, the consents have to be approved, and it needs a tool to extract data from devices. Depending on the company, there may be different data access policies in place, whereby it may not be possible to access the raw data of the wearable and lack of standardization and replicability of wearable raw data and analysis [39]. Therefore, the interoperation and standardization of data have to be considered to facilitate utilization of health technology in public health.

## Conclusion

In this study, it aims to address undetected geriatric depression in Thailand by exploring the link between smart device-collected data (such as physical activity and sleep patterns) and depression scores. A cohort study was conducted involving 177 elderly individuals from various regions and tracking their activity and sleep by Actigraph wGT3X-BT devices for a month with depression scoring every two weeks. Results showed associations between increased physical activity parameter (vector magnitude count per minute) and reduced depression scores, while sleep parameters (Wakefulness After Sleep Onset: WASO) showed an association only in univariate analysis. This suggests that there is a potential of using passive sensing data for mental health in Thailand by detecting physical activity as a key proxy of depression, but further research is needed to refine detection accuracy, predictive algorithms, and data integration. Utilizing this smart device-collected data in public health policies will require the development of interoperable systems to extract data from devices and standardization of data to ensure comparability.

## Supporting information

S1 Univariate analysis of between physical activity/sleep variables and demography, and PHQ-9 score. (DOCX)
