# Supplementary material for: Association between sleep and physical activity data, and depressive symptoms in Thai elderly
Source: PLoS One. 2025 Aug 14;20(8):e0329978. doi: 10.1371/journal.pone.0329978 (PMC12352767; doi:10.1371/journal.pone.0329978)
Supplement: S1 — (DOCX) [file pone.0329978.s001.docx]

**Table S1 Univariate analysis of between physical activity/sleep variables and demography, and PHQ-9 score (Round 2 and 3) adjusted with PHQ-9 (baseline)**

| **Independent/confounding variables** | **PHQ-9 score (Round 2)** | | | **PHQ-9 score (Round 3)** | | |
| --- | --- | --- | --- | --- | --- | --- |
|  | **Correlation coefficient** | **95% confident interval** | **P-value** | **Correlation coefficient** | **95% confident interval** | **P-value** |
| **Sex (vs female)** |  |  |  |  |  |  |
| male | 0.297 | 0.365 | 0.416 | 0.156 | 0.361 | 0.666 |
| **Province (vs BKK)** |  |  |  |  |  |  |
| North-Eastern | -0.905 | 0.403 | 0.026** | -0.014 | 0.412 | 0.973 |
| North-Eastern | -1.065 | 0.380 | 0.006** | -0.416 | 0.388 | 0.285 |
| Southern | -1.265 | 0.394 | 0.002** | -0.424 | 0.402 | 0.293 |
| Central | -1.062 | 0.404 | 0.009** | -0.254 | 0.412 | 0.539 |
| **Age** | 0.057 | 0.021 | 0.007** | 0.024 | 1.172 | 0.243 |
| **Education (vs illiterate)** |  |  |  |  |  |  |
| Primary education | -2.247 | 1.992 | 0.261 | -7.938 | 1.839 | <0.001** |
| Secondary education | -2.292 | 2.025 | 0.259 | -6.484 | 1.869 | <0.001** |
| Diploma | -2.372 | 2.020 | 0.242 | -7.756 | 1.865 | <0.001** |
| University and higher | -2.756 | 2.021 | 0.174 | -7.765 | 1.866 | <0.001** |
| **Occupation (vs Unemployed/Retired)** |  |  |  |  |  |  |
| Having occupation | -0.090 | 0.273 | 0.273 | -0.053 | 0.270 | 0.844 |
| **Income** | 0.000001 | 0.000004 | 0.865 | 0.000001 | 0.000004 | 0.750 |
| **Household members (vs alone)** |  |  |  |  |  |  |
| With parents/spouse/grandchildren | 0.343 | 0.471 | 0.466 | -0.639 | 0.463 | 0.169 |
| With mixed generations | 0.312 | 0.417 | 0.455 | -0.179 | 0.410 | 0.663 |
| **Marital status**  **(vs single/divorced/widowed)** |  |  |  |  |  |  |
| Married | -0.450 | 0.262 | 0.087* | -0.330 | 0.260 | 0.206 |
| **Current smoking (vs no)** |  |  |  |  |  |  |
| Yes | 2.102 | 0.988 | 0.035** | -0.389 | 0.991 | 0.695 |
| **Current alcohol used (vs no)** |  |  |  |  |  |  |
| Yes | -0.263 | 0.419 | 0.531 | -0.504 | 0.413 | 0.224 |
| **Current drug used (vs no)** |  |  |  |  |  |  |
| Yes (e.g, Cannabis) | 0.971 | 0.620 | 0.119 | 0.959 | 0.613 | 0.120 |
| **Comorbidity (physical illness) (vs none)** |  |  |  |  |  |  |
| Cardiovascular-related diseases | 0.031 | 0.370 | 0.932 | -0.156 | 0.367 | 0.670 |
| Orthopedic and Hormonal related diseases | 0.291 | 0.425 | 0.495 | -0.102 | 0.421 | 0.810 |
| **Comorbidity (mental illnesses) (vs none)** |  |  |  |  |  |  |
| Yes | 0.880 | 0.559 | 0.117 | 0.269 | 0.557 | 0.629 |
| **IADL (vs dependent)** |  |  |  |  |  |  |
| Independent | -0.502 | 0.462 | 0.278 | -0.914 | 0.453 | 0.045** |
| **Medicine affecting sleep and movement (vs no)** |  |  |  |  |  |  |
| Yes | -0.226 | 0.472 | 0.632 | -0.014 | 0.468 | 0.976 |
| **PA** |  |  |  |  |  |  |
| VM CPM | -0.000691 | 0.000332 | 0.039** | 0.000355 | 0.001314 | 0.793 |
| Step count | -0.000085 | 0.000046 | 0.063* | -0.000024 | 0.000218 | 0.913 |
| **Sleep** |  |  |  |  |  |  |
| WASO | -0.028 | 0.012 | 0.021** | -0.042 | 0.050 | 0.423 |
| SE | 0.062 | 0.039 | 0.107 | 0.047 | 0.200 | 0.820 |
| SFI | -0.009 | 0.018 | 0.633 | 0.139 | 0.093 | 0.167 |

**Note:** *confidence interval at 90%; **confidence interval at 95%
